# Evaluation of the Relative Citation Ratio Among Academic Orthopedic Hand Surgeons: A Novel Measure of Research Impact

**DOI:** 10.7759/cureus.25362

**Published:** 2022-05-26

**Authors:** John K McNamara, Matthew N Henderson, Suleiman Y Sudah, Robert D Faccone, Christopher R Michel, Christopher Dijanic, Mariano E Menendez, Jeremy B Ruskin

**Affiliations:** 1 Orthopedics, Rutgers Robert Wood Johnson Medical School, New Brunswick, USA; 2 Orthopedic Surgery, Monmouth Medical Center, Long Branch, USA; 3 Orthopedics, Alabama College of Osteopathic Medicine, Dothan, USA; 4 Orthopedic Surgery, Rush University Medical Center, Chicago, USA; 5 Orthopedic Surgery, Central Jersey Hand Surgery, Eatontown, USA

**Keywords:** publication count, bibliometric, hand surgery, fellowship, relative citation ratio

## Abstract

Background*: *Publication metrics such as article citation count and the Hirsch index (h-index) are used to evaluate research productivity among academic faculty. However, these bibliometric indices are not field-normalized and yield inaccurate cross-specialty comparisons. We evaluate the use of the relative citation ratio (RCR), a new field-normalized article-level metric developed by the National Institutes of Health (NIH), among academic orthopedic hand surgeons and analyze physician factors associated with RCR values.

Methods: A retrospective analysis was performed using the iCite database. Fellowship-trained orthopedic hand surgeons affiliated with accredited orthopedic surgery residency programs were included. Mean RCR, weighted RCR, and publication count were compared by sex, career duration, academic rank, and presence of additional degrees. Mean RCR represents the total number of citations per year of a publication divided by the average number of citations per year received by NIH-funded papers in the same field. Mean RCR serves as a measure of overall research impact. A value of 1.0 is the NIH-funded field-normalized standard. Weighted RCR is the sum of all article-level RCR scores and represents overall research productivity.

Results*: *A total of 620 academic orthopedic hand surgeons from 164 programs were included. These physicians produced highly impactful research with a median RCR of 1.27 (interquartile range [IQR] 0.86-1.66). Weighted RCR was associated with advanced degree, advanced academic rank, and longer career duration.

Conclusions*:* Fellowship-trained academic orthopedic hand surgeons produce highly impactful research. Our benchmark data can be used to assess grant outcomes, promotion, and continued evaluation of research productivity within the hand surgery community.

## Introduction

Objective measures of academic productivity are widely utilized by academic leadership and grant review panels in determining new faculty hires, promotion, tenure, and allocation of grant funds [1-3]. While the Hirsch index (h-index) has been traditionally used as the metric of choice for assessing research productivity [4,5], its use has been associated with many shortcomings [6,7]. The h-index combines frequency of publication and frequency of citation into a single numerical value, which often disadvantages younger authors with few but impactful publications and prioritizes quantity over quality [8]. Further, because the h-index is not field-normalized, its value is skewed by the size of the academic specialty, which limits the ability to make accurate cross-specialty comparisons [8,9]. For example, those publishing in a larger field, such as internal medicine, are likely to accrue a higher number of citations than publications within a niche subspecialty field. These limitations make the h-index an unsuitable metric for comparing authors that differ by field and career length [8,10-12].

The National Institutes of Health (NIH) developed a new field-normalized article-level metric called the relative citation ratio (RCR), which improves upon the weakness of the h-index and other popular bibliometric indices [13]. The RCR is calculated by dividing the total number of citations per year of a publication divided by the average citations per year received by NIH-funded papers in the same field [13]. Dynamic field-normalization through the use of a co-citation network of an article separates the RCR from traditional metrics and allows for a more accurate comparison of research impact across academic specialties [9,14]. Author-level derivatives of the RCR include mean RCR and weighted RCR and are calculated by taking the mean and sum, respectively, of all article-level RCR scores pertaining to a single researcher [13]. Because the mean RCR does not incorporate the total citation count of an article, its value is adjusted for time-dependent factors allowing for the comparison of research impact at different career stages. In contrast, the weighted RCR provides a metric for comparing the number of publications and article citations and may be used to determine overall research productivity. The RCR has been validated in a dataset of over 88,000 publications, demonstrating consistency with the expert opinion of research quality [13].

Herein, we conducted an RCR analysis of fellowship-trained academic orthopedic hand surgeons across the United States to provide benchmark data for RCR scores within the field and to identify correlates between these scores and various demographic groups, including sex, career duration, academic rank, and acquisition of a Doctor of Philosophy (PhD). The information presented in this study may serve as a more accurate gauge of research impact within the orthopedic hand community and can be used for self- and departmental evaluation as well as cross-specialty comparisons.

## Materials and methods

Departmental and faculty inclusion criteria

A comprehensive list of Accreditation Council for Graduate Medical Education (ACGME)-accredited orthopedic surgery residency programs in the United States was accessed in December 2021 (https://apps.acgme. org/ads/Public/Programs/Search). Individual departmental websites were visited to identify fellowship-trained orthopedic hand surgeons [Doctor of Medicine (MD) or Doctor of Osteopathic Medicine (DO)] employed as faculty. Exclusion criteria included hand surgery faculty members who were not fellowship-trained or those who completed residency training through general surgery or plastic surgery programs. Sex, the presence or absence of a Doctor of Philosophy degree (PhD), academic rank, and career longevity (as determined by residency start year) were obtained using physician profiles on departmental websites or via publicly available outlets such as Doximity and LinkedIn. Academic ranks included assistant, associate, and full professor. Clinical assistant professors, instructors, and lecturers were considered assistant professors. Staff physicians, private practice physicians, or faculty not otherwise specified were listed as “Other.” Residency start years were obtained to categorize faculty into the following groups: ≤1980, 1981-1990, 1991-2000, 2001-2010, and >2010.

Bibliometric analysis

An individual publication’s RCR is defined as the total number of citations per year for that publication divided by the average field-specific citations per year for all NIH-funded publications in that same field. The specific research field an article is classified into is defined by its co-citation network, which is the body of publications cited alongside the article of interest. An RCR of 1.0 represents the field-normalized benchmark for NIH-funded research. Author-level RCR scores (mean and weighted RCR) are calculated from the aggregate article-level RCR scores for all publications pertaining to that author. An author’s mean RCR is the statistical average of all RCR scores for each of their publications and reflects the overall research impact. An author’s weighted RCR is the sum of all RCR scores for each of their publications and reflects overall research productivity. Orthopedic hand surgery faculty members were individually indexed using the iCite database website (https://icite.od.nih.gov/). The iCite database includes PubMed-indexed articles from 1980 to the present. Non-original research articles such as editorials, reviews, and meeting abstracts were excluded from the analysis. The total number of publications, mean RCR score, and weighted RCR score were recorded for each author on December 15, 2021.

Statistical analysis

The mean RCR, weighted RCR, and total publication count for each faculty member were recorded and compared by sex, degree, academic rank, and career longevity as defined by residency start date. The Wilcoxon rank-sum test was used for two-group analyses. Analysis of variance (ANOVA) testing was used for comparisons between three or more subgroups. Statistical significance was achieved at p <0.05. The data herein are presented as the median and interquartile range (IQR) (in addition to the mean and standard deviation) to account for outliers of the mean and weighted RCR scores.

## Results

A total of 620 fellowship-trained academic orthopedic hand surgeons were included in this study (Table 1). The majority of faculty were male, 512 (82.6%), and 18 (2.9%) had a PhD. Overall, RCR scores were high but variable with a median RCR of 1.27 (IQR 0.86-1.66) (Table 2) and a median weighted RCR of 13.12 (IQR 4.27-37.61) (Table 3). The median total number of publications per physician was 11.00 (IQR 4.00-32.00) (Table 4).

**Table 1:** Demographics for academic hand faculty members ^a^Assistant professor includes clinical assistant professor, instructor, and lecturer. ^b^“Other” indicates clinical instructors, staff physicians, or other faculty not otherwise specified.

Characteristic	No.	%
Sex		
Female	108	17.4
Male	512	82.6
PhD Degree		
No	602	97.1
Yes	18	2.9
Academic Ranking		
Assistant Professor^a^	330	53.2
Associate Professor	119	19.2
Professor	111	17.9
Other^b^	60	9.7
Residency Start Year		
≤1980	51	8.2
1981-1990	97	15.6
1991-2000	137	22.1
2001-2010	210	33.9
>2010	125	20.2

**Table 2:** Mean RCR by sex, PhD acquisition, academic ranking, and residency start year ^a^Assistant professor includes clinical assistant professor, instructor, and lecturer. ^b^“Other” indicates clinical instructors, staff physicians, or other faculty not otherwise specified. RCR, relative citation ratio; PhD, Doctor of Philosophy.

Characteristic	No.	Mean	SD	Median	25th Percentile	75th Percentile	p-Value
Sex							
Female	108	1.43	0.96	1.25	0.92	1.67	0.68
Male	512	1.38	1.19	1.28	0.85	1.66	
PhD Degree							
No	602	1.39	1.17	1.26	0.85	1.66	0.97
Yes	18	1.40	0.38	1.46	1.18	1.59	
Academic Ranking							
Assistant Professor^a^	330	1.43	1.37	1.27	0.83	1.71	0.38
Associate Professor	119	1.40	0.97	1.28	0.95	1.60	
Professor	111	1.42	0.77	1.34	0.98	1.66	
Other^b^	60	1.15	0.71	1.08	0.58	1.62	
Residency Start Year							
≤1980	51	1.24	0.71	1.09	0.86	1.68	0.26
1981-1990	97	1.57	1.59	1.34	0.88	1.70	
1991-2000	137	1.28	0.91	1.14	0.74	1.60	
2001-2010	210	1.37	0.89	1.28	0.86	1.64	
>2010	125	1.48	1.48	1.40	0.96	1.70	
Total	620	1.39	1.15	1.27	0.86	1.66	

**Table 3:** Weighted RCR by sex, PhD acquisition, academic ranking, and residency start year ^a^Assistant professor includes clinical assistant professor, instructor, and lecturer. ^b^“Other” indicates clinical instructors, staff physicians, or other faculty not otherwise specified. Asterisks denote statistical significance as determined by p-value <0.05. RCR, relative citation ratio; PhD, Doctor of Philosophy.

Characteristic	No.	Mean	SD	Median	25th Percentile	75th Percentile	p-Value^*^
Sex							
Female	108	24.07	41.44	8.62	3.68	26.52	0.048^*^
Male	512	35.34	56	14.55	4.43	41.08	
PhD Degree							
No	602	32.38	53.30	12.21	4.19	35.06	0.007^*^
Yes	18	66.91	63.84	53.89	23.40	83.84	
Academic Ranking							
Assistant Professor^a^	330	18.90	35.30	7.80	3.11	21.98	<0.001^*^
Associate Professor	119	35.62	37.47	24.84	7.85	43.21	
Professor	111	85.94	85.17	65.61	26.40	102.38	
Other^b^	60	11.35	17.67	4.06	2.03	9.80	
Residency Start Year							
≤1980	51	44.47	59.39	15.46	5.01	82.55	<0.001^*^
1981-1990	97	48.37	74.96	19.01	5.02	58.49	
1991-2000	137	37.57	60.15	17.34	4.06	41.06	
2001-2010	210	28.80	40.24	12.25	4.41	33.45	
>2010	125	20.31	39.67	8.86	3.60	24.17	
Total	620	33.38	52.89	13.12	4.27	37.61	

**Table 4:** Total number of publications by sex, PhD acquisition, academic ranking, and residency start year ^a^Assistant professor includes clinical assistant professor, instructor, and lecturer. ^b^“Other” indicates clinical instructors, staff physicians, or other faculty not otherwise specified. Asterisks denote statistical significance as determined by p-value <0.05. PhD, Doctor of Philosophy.

Characteristic	No.	Mean	SD	Median	25th Percentile	75th Percentile	p-Value^*^
Sex							
Female	108	20.07	31.23	8	4	20	0.075
Male	512	27.54	41.02	12	4.75	33	
PhD Degree							
No	602	25.38	38.87	10	4	29.75	0.002*
Yes	18	54.83	51.90	48.50	20.25	66/75	
Academic Ranking							
Assistant Professor^a^	330	14.64	23.72	7	4	15	<0.001*
Associate Professor	119	29.81	31.38	22	9	38.50	
Professor	111	65.66	62.08	54	23.50	82	
Other^b^	60	10.03	12.79	5.00	3.00	9.25	
Residency Start Year							
≤1980	51	35.51	51.78	15	6	50.50	<0.001*
1981-1990	97	35.81	51.66	12	4	43	
1991-2000	137	30.95	46.08	14	5	38	
2001-2010	210	23	29.77	12	4.25	26	
>2010	125	15.30	23.99	8	4	16	
Total	620	26.24	39.57	11	4	32	

Sex

Female hand surgeons had a median RCR of 1.25 (IQR 0.92-1.67) compared to 1.28 (IQR 0.85-1.66) for males. This finding was not statistically significant (p = 0.682). There was also not a statistically significant difference between the median total number of publications among female and male faculty members [8.00, IQR 4.00-20.00 vs 12.00, IQR 4.75-33.00) (p = 0.075). There was a statistically significant difference in weighted RCR (p = 0.048), with males having a median weighted RCR of 14.55 (IQR 4.43-41.08) and females having a median weighted RCR of 8.62 (IQR 3.68-26.52).

Career duration

Increased career longevity (defined by residency start date) had a significant impact on total publication count (p < 0.001) and weighted RCR scores (p < 0.001). Those with the longest career durations (residency start date ≤1980) had the highest median total number of publications with 15.00 (IQR 6.00-50.50). The highest weighted RCR was seen among physicians who began residency between 1981 and 1990, with a median value of 19.01 (IQR 5.02-58.49). Those with the shortest career durations had the lowest median total number of publications with 8.00 (IQR 4.00-16.00) and the lowest median weighted RCR scores with 8.86 (IQR 3.60-24.17). No significant association between career longevity and median RCR was found (p = 0.26).

Academic rank

The most common academic rank in this sample was assistant professor (53.2%), followed by associate professor (19.2%) and professor (17.9%). The remaining 9.7% of physicians were categorized as “Other.”

There was a significant association between total publications (p < 0.001) and weighted RCR (p < 0.001) with academic rank. There is not a significant relationship between academic rank and mean RCR (p = 0.38). Full professors were the most productive academic rank subgroup, with a median RCR of 1.34 (IQR 0.98-1.66) and a median weighted RCR of 65.61 (IQR 26.40-102.31).

PhD faculty had a significantly greater median total publication count (48.50, IQR 20.25-65.75 vs 10.00, IQR 4.00-29.75; p = 0.002) and weighted RCR (53.89, IQR 23.40-83.84 vs 12.21, IQR 4.19-35.06; p = 0.007) when compared to non-PhD faculty. There was no statistically significant difference between the median PhD and non-PhD RCR scores (1.46, IQR 1.18-1.59 vs 1.26, IQR 0.85-1.66; p = 0.971).

## Discussion

Our analysis of the RCR publication metric shows that fellowship-trained academic orthopedic hand surgeons produce highly impactful research, as evidenced by the high median RCR value relative to the NIH standard RCR value of 1.0. The information presented in this study may serve as a more accurate measure of research productivity and impact within the academic hand surgery community and can be used for self- and departmental evaluation as well as cross-specialty comparisons.

The median RCR for all PubMed-listed publications indexed on the iCite database is 0.37 (range, 10th-90th percentile, 0.00-2.24), whereas the median RCR for all NIH-funded publications is 1.00 (range, 10th percentile to 90th percentile, 0.20-3.81). In our study, the median RCR was 1.27 (IQR 0.86-1.66), which suggests that publications of academic orthopedic hand surgeons are highly influential when compared to PubMed-listed and NIH-funded publications. Specifically, this RCR value falls within the 70th-80th percentile of RCR scores among all publications within the iCite database, and within the 50th-60th percentile among all NIH-funded publications. Rock et al. recently evaluated the use of the RCR among 1299 academic radiation oncologists from 75 institutions, and reported a median RCR of 1.32 (IQR 0.87-1.94) [14]. Likewise, Reddy et al. evaluated this metric within the field of academic neurosurgery [9]. Overall, a median RCR of 1.37 (IQR 0.93-1.97) was reported among 1687 neurosurgery faculty members from 125 institutions. Our data suggest that research impact among academic orthopedic hand surgeons is comparable to that of academic faculty members in other medical specialties, including neurosurgery, radiation oncology, and ophthalmology [9,14,15]. 

Each subgroup analyzed was found to have an RCR value greater than 1.0. Specifically, there was a statistically significant association between academic rank and longer career duration with weighted RCR scores. Longer career duration, as determined by residency start date, had a statistically significant association with total publication count (p < 0.001) and weighted RCR score (p < 0.001), suggesting that more experienced faculty members demonstrate greater overall research productivity. This relationship is unsurprising, seeing as an individual’s weighted RCR score is directly related to their total publication count. Thus, the weighted RCR serves as an important measure of overall research productivity over time. However, we found no statistically significant relationship between career duration and median RCR score (p = 0.26), suggesting that research impact has remained similar among faculty members over time.

In contrast, significant associations were seen between career longevity and median RCR among academic radiation oncologists and neurosurgeons [9,14]. Potential explanations for the higher median RCR scores among older neurosurgeons include advancing academic rank, greater individual experience, and increased research funding [14]. Reddy et al. showed that 75% of the neurosurgeons within the longest career subgroup, defined as beginning residency in 1980 or earlier, had achieved the rank of full academic professor [14]. Similarly, we found that nearly half of the orthopedic hand surgeons (49%, 25 of 51) within the longest career subgroup were full academic professors. The parallel between advancing academic rank and increasing career longevity may account for the significant associations found between these subgroups and median publication count and weighted RCR. While faculty members of higher rank had greater overall research productivity, we found no statistically significant relationship with median RCR scores. This indicates that publication-level impact may be independent of the author’s academic position.

While sex-specific analysis revealed no significant impact on the mean RCR (p = 0.682) between male and female orthopedic hand surgeons, a statistically significant difference in weighted RCR was found in favor of males (14.55 vs 8.62, p = 0.048). This suggests that female orthopedic hand surgeons may produce fewer but similarly impactful studies when compared to their male counterparts. Similarly, no significant differences in median RCR were seen among female neurosurgeons [14] or female radiation oncologists [9] while males were found to have significantly higher weighted RCR scores in both specialties. However, it is important to acknowledge that the majority of hand surgeons (82.6%), neurosurgeons (91%), and radiation oncologists (69.1%) evaluated were male [9,14]. As sex representation becomes more equalized in orthopedic surgery, these differences are likely to change over time.

In addition, we found that PhD faculty demonstrate significantly greater publication counts (48.5 vs 10; p = 0.002) and weighted RCR (53.89 vs. 12.21, p = 0.007) compared to non-PhD faculty. However, there was no significant difference in median RCR between groups (1.46 vs 1.26; p = 0.971). This trend suggests that while PhD faculty may have greater overall research productivity, those without this advanced degree seem to produce equally impactful studies within the field of orthopedic hand surgery. However, as only 2.9% (18 of 620) of faculty had a PhD, these conclusions may be limited by an underpowered sample.

While the RCR demonstrates many strengths compared to other bibliometric indices, it is not without limitations. Like other bibliometric indices, the RCR is unable to differentiate authorship contribution and, thus, may not accurately reflect research impact based on differing levels of author seniority. Furthermore, although the RCR allows for greater interdisciplinary comparison, it is a relatively novel metric, and its practical implications have not been fully elucidated. Other limitations of our study include limited generalizability and potential errors in data collection. Our study cohort included only fellowship-trained orthopedic hand surgery faculty. However, non-fellowship trained physicians as well as those who completed general surgery or plastic surgery residency training may serve on faculty within orthopedic hand surgery programs. In addition, the iCite website does not differentiate between individuals with the same name, which presents a potential source of error. Potential errors were mitigated by searching authors' middle initials and reviewing individual publication titles. The iCite website also only includes PubMed articles published from 1980 to the present, which may underestimate the RCR of researchers with publications prior to 1980. Furthermore, since the specific research field into which a particular article is classified is dependent on its co-citation network, it is possible for articles to be misclassified, and thus the RCR for that article may not be accurate. 

An additional factor to take into consideration when evaluating the RCR as a metric is the effect of social media use among medical professionals. Social media platforms, such as Twitter and Instagram, have been employed as networking tools within a variety of medical communities and have enabled individuals within and across specialties to connect with one another. Individual authors may use these connections or their existing social media presence to enhance their number of citations, whether by promoting their own publications or by promoting the journals in which their work has been published, regardless of that journal's traditional impact factor. In this manner, the dynamic nature of social media's use within academic medicine may further confound the interpretation of the RCR in ways that are yet to be determined.

## Conclusions

The RCR and its derivatives serve as novel metrics that more accurately reflect research impact and are less encumbered by the drawbacks of traditionally used bibliometrics. Our study shows that fellowship-trained orthopedic hand surgeons serving as academic faculty members are highly productive and generate impactful research when compared to physicians in other specialties and to the general scientific community. The information gleaned from this study can be used as a standard to evaluate the improvement of grant outcomes, promotion, education, and continued assessment of research productivity and impact within the hand surgery community. Additional evaluation of the RCR across other specialties and topics is required to solidify its use as a measure of research productivity and impact. 
